# Effects of Horticultural Therapy on Asian Older Adults: A Randomized Controlled Trial

**DOI:** 10.3390/ijerph15081705

**Published:** 2018-08-09

**Authors:** Kheng Siang Ted Ng, Angelia Sia, Maxel K. W. Ng, Crystal T. Y. Tan, Hui Yu Chan, Chay Hoon Tan, Iris Rawtaer, Lei Feng, Rathi Mahendran, Anis Larbi, Ee Heok Kua, Roger C. M. Ho

**Affiliations:** 1 Department of Psychological Medicine, Yong Loo Lin School of Medicine, National University of Singapore, Singapore 119228, Singapore; ted.ng@duke-nus.edu.sg (K.S.T.N.); chan_hui_yu@u.nus.edu (H.Y.C.); phctanch@nus.edu.sg (C.H.T.); iris_rawtaer@nuhs.edu.sg (I.R.); pcmfl@nus.edu.sg (L.F.); pcmrathi@nus.edu.sg (R.M.); pcmkeh@nus.edu.sg (E.H.K.); 2 Centre for Urban Greenery and Ecology Research, National Parks Board, Singapore 259569, Singapore; angelia_sia@nparks.gov.sg; 3 Horticulture and Community Gardening Division, National Parks Board, Singapore 259569, Singapore; maxel_ng@nparks.gov.sg; 4 Singapore Immunology Network, Agency for Science, Technology and Research, Singapore 138648, Singapore; crystal_tan@immunol.a-star.edu.sg (C.T.Y.T.); anis_larbi@immunol.a-star.edu.sg (A.L.); 5 Department of Pharmacology, Yong Loo Lin School of Medicine, National University of Singapore, Singapore 119228, Singapore; 6 Department of Psychological Medicine, National University Hospital, Singapore 119074, Singapore

**Keywords:** horticultural therapy, RCT (randomized controlled trial), cytokines, IL-6, positive relationship

## Abstract

The effect of horticultural therapy (HT) on immune and endocrine biomarkers remains largely unknown. We designed a waitlist-control randomized controlled trial to investigate the effectiveness of HT in improving mental well-being and modulating biomarker levels. A total of 59 older adults was recruited, with 29 randomly assigned to the HT intervention and 30 to the waitlist control group. The participants attended weekly intervention sessions for the first 3 months and monthly sessions for the subsequent 3 months. Biological and psychosocial data were collected. Biomarkers included IL-1β, IL-6, sgp-130, CXCL12/SDF-1α, CCL-5/RANTES, BDNF (brain-derived neurotrophic factor), hs-CRP, cortisol and DHEA (dehydroepiandrosterone). Psychosocial measures examined cognitive functions, depression, anxiety, psychological well-being, social connectedness and satisfaction with life. A significant reduction in plasma IL-6 level (*p* = 0.02) was observed in the HT intervention group. For the waitlist control group, significant reductions in plasma CXCL12 (SDF-1α) (*p* = 0.003), CXCL5 (RANTES) (*p* = 0.05) and BDNF (*p* = 0.003) were observed. A significant improvement in social connectedness was also observed in the HT group (*p* = 0.01). Conclusion: HT, in reducing plasma IL-6, may prevent inflammatory disorders and through maintaining plasma CXCL12 (SDF-1α), may maintain hematopoietic support to the brain. HT may be applied in communal gardening to enhance the well-being of older adults.

## 1. Introduction

During the process of normal aging, there are neurobiological changes such as the loss of neurons [1], and psychosocial changes that negatively affect cognition and social connectedness [2]. Correspondingly, horticultural therapy (HT) is becoming an increasingly popular non-pharmacological method to improve the well-being of older adults [3]. HT is relatively easy to implement, and this has made it a popular preventive measure in enhancing the health of older adults. From a medical perspective, the positive effects of garden settings on patients with mental disorders [4] and hospitalized war veterans have been shown [5]. The therapeutic effects of HT include social engagement [2,3] and preventing cognitive decline [3] and depression [6]. HT was found to improve the physical and mental well-being of patients with chronic musculoskeletal pain [7], as well as patients in a cardiac rehabilitation ward [8]. 

A recent study by Li et al. (2010) reported increases in the activity of natural killer (NK) cells and its enzymes after a forest bathing trip [9]. The authors speculated that decreased stress hormones may mediate this phenomenon. Horticultural gardens in a hospital setting have been reported to increase well-being and social connectedness among patients with brain damage as well [10]. One of the core objectives of HT is to promote social support and sharing amongst the participants. Noone et al. (2015) demonstrated that communal gardening with its collaborative features, may instil a sense of belonging to the community, hence improving social integration [11]. 

Although there have been numerous studies on HT, a systematic review of randomized controlled trials (RCTs) on HT concluded that the studies conducted so far were relatively low quality [12]. In particular, descriptions of the study designs and statistical analyses had mostly been omitted. Also, most of the RCTs employing HT have focused mainly on severe mental disorders, such as patients with dementia, depression, and schizophrenia. Although all studies done so far have showed significant improvements in their respective primary outcome measures, a few major limitations were noted. Amongst the limitations was the small sample size of most HT studies, the short intervention duration and the lack of a control group [12]. These issues limit the generalizability and applicability of the findings and call into question the validity of the results.

It has been postulated that increased levels of IL-6 and IL-1β are involved in the pathogenesis of Alzheimer’s disease and major depressive disorders [13]. On the other hand, the presence of soluble glycoprotein 130 (sgp130), which inhibits IL-6 trans-signalling [14], may modify the association between elevated interleukin 6 soluble receptor (sIL6R) levels and myocardial infarction [15]. The chemokine CXCL12 (SDF-1α) controls the functions of bone marrow-derived stem cell functions and neurogenesis and there are decreased level of CXCL12 in early Alzheimer’s disease [16]. Lower levels of CXCL-5 (RANTES) have been observed in patients suffering from dementia, when compared to healthy individuals. Higher levels of BDNF (brain-derived neurotrophic factor), which has been shown to improve synaptic plasticity and long-term memory, may decrease the risk of dementia [17]. Dementia and depression in older adults are also associated with changes in hormone levels, such as cortisol and dehydroepiandrosterone (DHEA) levels [18]. Despite the important roles of these biomarkers, the specific effects of HT on the above-mentioned biological markers remain largely unknown.

In view of this, Lin et al. suggested that measures of biomarkers should be incorporated in future studies [4]. This proposition was supported by the findings of Mao et al. which showed that forest bathing reduced cardiovascular disease-related biomarkers, one of which was IL-6 [19]. Additionally, Ochiai et al. showed that forest bathing reduced blood pressure (BP), urinary adrenaline, and both serum and salivary cortisol [20,21]. In this HT-randomized controlled trial, we aimed to measure the changes in the levels of pro-inflammatory cytokines, interleukin (IL)-6, IL-1β, soluble glycoprotein 130 (sgp130), chemokines, C-X-C motif chemokine 12 (CXCL12), also known as stromal cell-derived factor 1 alpha (SDF-1α), C-C motif ligand 5 (CCL-5), also known as regulated on activation, normal T-cell expressed and secreted (RANTES) and BDNF, all of which exert pleiotropic effects on the central nervous system and are key modulators of the immune and nervous system. Based on the findings of past studies, we also hypothesized that increased social connection would be observed in the HT group after intervention. 

## 2. Materials and Methods 

### 2.1. Participants

This trial began with the Ethics Board’s approval with NUS-IRB (approval number: B-15-016). It was also registered with Clinicaltrials. gov, identifier: NCT02495194 (https://clinicaltrials.gov/ct2/show/NCT02495194). The participants were 59 older adults between the ages of 61 and 77 (mean = 67.1 years, SD = 4.31) recruited from a neighbourhood in the western region of Singapore. All the participants were screened based on the inclusion and exclusion criteria. That is, the participants should be aged between 60 to 85 years old, able to provide informed consent and have a minimum Montreal Cognitive Assessment (MoCA) score of 22. They must not have current or past histories of severe medical or psychiatric disorders. They must not be undergoing any concurrent therapy, and should have no significant visual or hearing impairments or upper and lower limb motor difficulties. Subsequently, qualified participants were randomized into either the HT intervention group or waitlist control group.

### 2.2. The Interventions

The HT intervention was designed by an experienced instructor. The outdoor sessions were conducted at a few selected parks/gardens and a nature reserve in Singapore. The intervention comprised a total of 15 sessions, with activities ranging from indoor gardening, growing, maintaining and harvesting vegetables and herbs to guided walks in the various parks (Table 1). The duration of each session was approximately one hour. They were conducted weekly for three months, and then monthly for the subsequent three months. To be ethical and fair to the participants in both groups and to enable all the participants to experience HT, a waitlist control design was employed in place of an active control design. The waitlist control group began HT only after the HT intervention group had completed all the interventions. 

### 2.3. Outcome Measurements

Both HT and control groups had psychosocial assessments and venepunctures taken at the same time-points, namely, at the start of the study (baseline), three months and six months after the HT intervention group received treatment. The control group started its first session only after the completion of all the above-mentioned biopsychosocial assessments. Thereafter, there were no further assessments on the waitlist control group.

### 2.4. Biological Measures

To measure the biomarkers, ten millilitres of fasting venous blood were withdrawn from each participant between 8.30 a.m. and 9.30 a.m. at the research centre. The blood was sent to the laboratory within three hours after the first venepuncture. Subsequently, the whole blood samples were centrifuged at 1650 *g* for 25 min at room temperature to obtain the plasma. All the blood and plasma samples from both groups at the three time-points were stored at −80 °C. The nine plasma biomarkers were examined using commercially available enzyme-linked immunosorbent assay (ELISA) kits, namely, IL-6, IL-1β (ThermoFisher, Waltham, MA, USA), CXCL12/SDF-1α, CXCL5/RANTES (R&D Systems Inc., Minneapolis, MN, USA), BDNF (Promega Corporation, Madison, WI, USA), DHEA (CUSABIO, Houston, TX, USA), cortisol, hs-CRP (Tecan, Männedorf, Switzerland), and sgp130 (RayBiotech Inc., Norcross, GA, USA). Assays for each biomarker were run on the same day to avoid batch effect.

### 2.5. Psychosocial Measures

The Montreal Cognitive Assessment (MoCA) was used to assess the cognitive function of the participants. We used the Zung Self-Rating Depression Scale (SDS), and the Zung Self-Rating Anxiety Scale (SAS) to assess psychopathology and the Ryff’s Scales of Psychological Well-Being, Friendship Scale and Satisfaction with Life Scale to assess the psychosocial well-being of the participants. Detailed descriptions of the measures can be found in our protocol paper [22].

### 2.6. Statistical Analyses

All data were collected and analysed from 2015 to 2017. The biological and psychosocial measurements were expressed as mean ± standard deviation (SD). Baseline variables were compared using Student’s *t*-test, chi-square or Fisher’s exact tests. A general linear model using repeated-measured analysis of variance (rANOVA) was employed to examine the difference between the active treatment and waitlist control groups. The three time-points were entered as a within-participants factor (indicated as “time”) while the two groups of participants were entered as a between-participants factor (indicated as “group”). When the rANOVA indicated significant interaction between group and time (between-group difference), or a significant main effect of time (within-group difference), post-hoc analyses of paired T-tests were conducted to determine the pair of data that differed significantly. A two-tailed *p*-value of <0.05 was considered as statistically significant. Alpha values of the paired T-tests were adjusted for using Bonferroni’s correction. Analyses and construction of the graphs and figures were performed using the Statistical Package for the Social Sciences (SPSS) version 23.0 (IBM, Armonk, NY, USA) and GraphPad Prism version 6 (GraphPad Software, San Diego, CA, USA), respectively. Mean imputation of data was performed to replace loss-to-follow-up missing values, hence enabling intention-to-treat analyses. Log- or squared-transformations were employed for highly skewed data.

## 3. Results

### 3.1. Baseline Data

**Figure 1:**
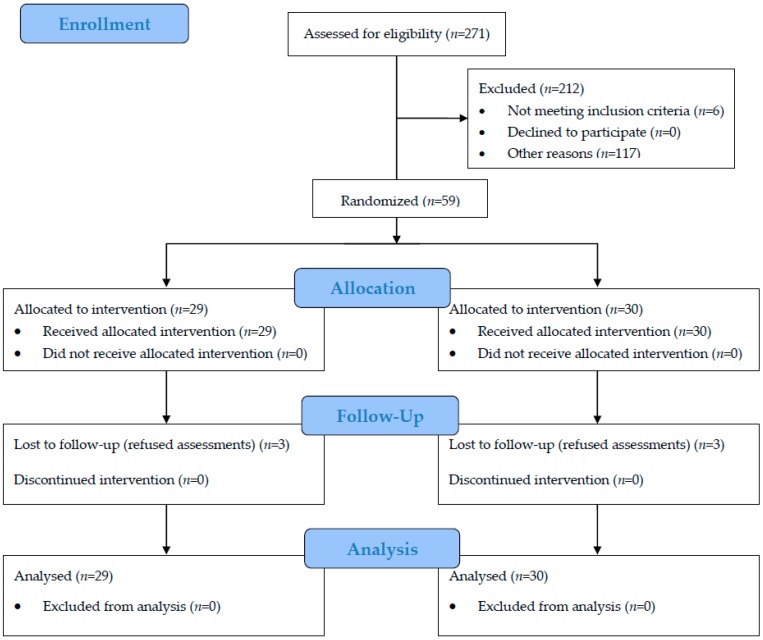
CONSORT flow diagram for HT-randomized controlled trial (RCT).

Table 2 shows the baseline characteristics of the HT and waitlist control groups. There were no significant differences in terms of demographics and psychosocial assessments between the two groups (*p* > 0.05). Figure 1 shows the flow of the participants throughout the intervention period.

### 3.2. Effects of HT Intervention on Biological Markers

#### 3.2.1. Plasma IL-6 Levels

**Figure 2:**
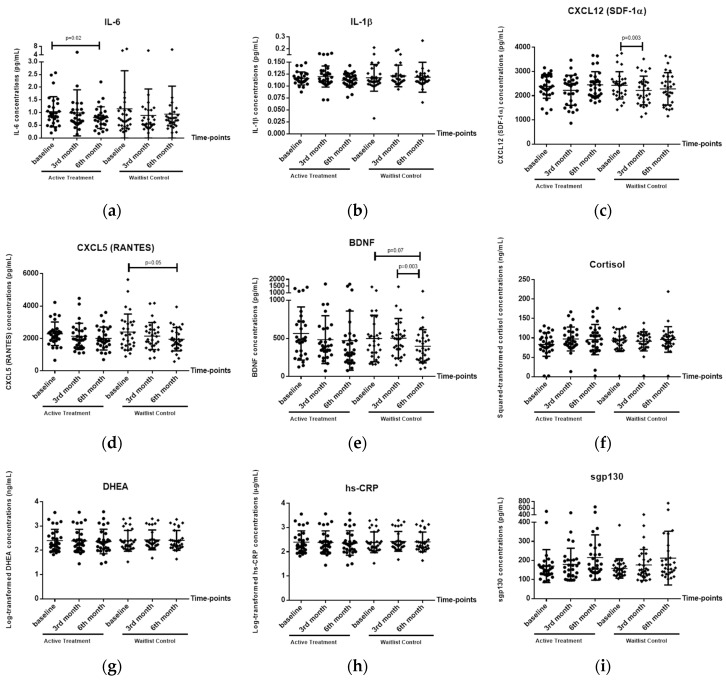
Changes of plasma biomarker levels in horticultural therapy (HT) and waitlist control groups. (**a**): IL-6; (**b**): IL-1β; (**c**): CXCL12 (SDF-1α); (**d**): CXCL5 (RANTES); (**e**): BDNF (brain-derived neurotrophic factor); (**f**): Cortisol; (**g**): DHEA (dehydroepiandrosterone); (**h**): hs-CRP; (**i**): Sgp-130.

There was a trend suggesting that time had a significant effect on IL-6 levels (*p* = 0.095) (Table 3). Post hoc analysis revealed a significant decrease in plasma IL-6 levels from baseline to 6-months (−0.23 pg/mL, 95% CI = −0.40 to −0.07, *p* = 0.02) in the HT group (Figure 2a). There was no significant change observed in the waitlist control group. No significant effects of group and group × time interactions were found on IL-6 levels.

#### 3.2.2. Plasma CXCL12 (SDF-1α) Levels 

Time had a significant effect on CXCL12 (SDF-1α) levels (*p* = 0.007) (Table 3). Post hoc analysis revealed a significant decrease in plasma CXCL12 levels from baseline to 3-months (−210.09 pg/mL, 95% CI = −330.73 to −89.46, *p* = 0.003) in the waitlist control group (Figure 2c). There was no significant change observed in the HT group. No significant effects of group and group × time interactions were found on CXCL12 (SDF-1α) levels.

#### 3.2.3. Plasma CXCL5 (RANTES) Levels

Time had a significant effect on CXCL5 (RANTES) levels (*p* = 0.005) (Table 3). Post hoc analysis revealed a borderline significant decrease in plasma CXCL5 levels from baseline to 6-months (−440.86 pg/mL, 95% CI = −799.79 to −81.93, *p* = 0.05) in the waitlist control group (Figure 2d). There was no significant change observed in HT group participants. No significant main effects of group and group × time interactions were found on CXCL5 (RANTES) levels.

#### 3.2.4. Plasma BDNF Levels

Time had a significant effect on BDNF levels (*p* = 0.02) (Table 3). Post hoc analysis indicated a decreasing trend in plasma BDNF levels from baseline to 6-months in the HT group (−101.46 pg/mL, 95% CI= −14.44 to −188.49 *p* = 0.07). A significant decrease in plasma BDNF levels from 3-months to 6-months (−104.83 pg/mL, 95% CI = −48.12 to −161.55, *p* = 0.003) in the waitlist control group was also observed (Figure 2e). There was no significant change observed in the HT group across all the time-points. No significant effects of group and group × time interactions were found on BDNF levels.

#### 3.2.5. Plasma IL-1β, Cortisol, DHEA, hs-CRP and Sgp-130 Levels

Based on the rANOVA results, there were no significant effects of time, effects of group or time × group interactions on the levels of these four biomarkers: IL-1β, cortisol, DHEA and hs-CRP. Hence, no post hoc analyses were performed. There was a significant effect of time on Sgp-130 levels (*p* = 0.01) (Table 3). Post hoc analyses were performed and no significant changes in either the HT or waitlist control groups were found (Figure 2i). 

### 3.3. Effects of HT Intervention on Psychological Well-Being

#### 3.3.1. Positive Relations with Others 

There was a significant time × group interaction on the sub-scale of the Ryff’s Scales of Psychological Well-Being, positive relations with others (*p* < 0.001) (Table 4). Post hoc paired T-test analyses were conducted to examine the changes. In HT participants, the mean score of positive relations with others, a sub-scale of the Ryff’s Scales of Psychological Well-Being, was significantly improved 6 months after study entry. Relative to baseline, the mean scores of positive relations with others was significantly increased by 2.14 points (95% CI: 0.52 to 3.76) in HT participants (*p* = 0.001). On the other hand, the change in the control group after 6 months was not significant (*p* = 0.31), with the mean scores reduced by −0.7 points (95% CI: −2.09 to 0.69). 

#### 3.3.2. Cognitive Function, Depression, Anxiety and Psychological Well-Being

There were significant effects of time on MoCA (*p* = 0.004) and SAS scores (*p* = 0.03). There were no significant interactions between time and study groups nor major effects of time or study group in other psychometric measures (Table 4). No significant post-hoc analyses were found for MoCA. SAS scores increased in both groups from baseline to 6-months, in the treatment group from 26.00 (4.88) to 36.32 (4.91), (*p* < 0.001), while in the control group from 25.79 (5.07) to 34.21 (3.69), (*p* < 0.001) (Table 4). 

## 4. Discussion

Using an RTC design, preliminary effects on the biological and psychological benefits of HT were explored. A significant decrease in the mean plasma IL-6 level was observed in the HT group but not in the waitlist control group. On the other hand, significant decreases in plasma CXCL5 (RANTES), CXCL12 (SDF-1α) and BDNF were observed in the waitlist control group but not in the HT group. The changes in these biomarkers suggest that engagement in HT may elicit positive biological changes, such as the reduction of inflammation and the ability to maintain hematopoietic brain support and neuroprotection, hence decreasing the risk of psychiatric morbidities, particularly dementia. Similarly, the analyses showed a significant increase in the mean scores of the psychosocial scale, positive relations with others, in the HT group, but not the control group. There were no significant changes in plasma IL-1β, cortisol, DHEA, hs-CRP and Sgp-130 levels and other psychosocial scales in both the HT and control groups.

The significant reduction in plasma IL-6 levels after receiving the HT intervention suggests that HT may attenuate heightened inflammation in older adults. High IL-6 levels have been associated with a variety of inflammation-associated diseases including rheumatoid arthritis [13], asthma [23] and obesity [24]. In contrast, lower IL-6 levels are associated with higher scores in positive relationships. From a social perspective, low socioeconomic status is shown to be related to higher IL-6 plasma levels in older adult women [25]. From a psychiatry perspective, high levels of IL-6 are associated with impairment in encoding and recall in healthy and depressed older adults [26]. IL-6 is an important marker of leukoaraiosis and contributes to silent cerebral infarction [27] which could result in dementia [28,29] and depression. From a physical health perspective, older adult women with high pro-inflammatory status present with lower relative muscle strength [30]. A higher level of IL-6 is associated with various medical conditions in older adults, including delirium after non-cardiac surgery [31], colorectal carcinoma [32] and poor prognosis in older adult patients with chronic lymphocytic leukemia [33]. A higher level of IL-6 is also associated with a significantly greater risk of disability [34] and mortality [35] in older adults. Correspondingly, we showed that HT reduced IL-6 levels and this reduction may play a role in preventing various adverse health outcomes.

The significant reduction in plasma CXCL12 (SDF-1α) levels in the control group suggests deficient hematopoietic brain support which may lead to the deterioration of blood supply to the brain [16]. Correspondingly, a reduction in CXCL12 (SDF-1α) levels was found in patients with Alzheimer’s disease [36]. Downregulation of CXC12 (SDF-1α) mRNA was also found in the hippocampus of a mouse model of Alzheimer’s disease with cognitive deficits [37]. Low plasma CXC12 (SDF-1α) levels were significantly correlated with higher tau protein levels in the cerebrospinal fluid (CSF) [16]. Cerebrovascular degeneration and hypoperfusion of the brain often affect the severity of cognitive decline in patients with prodromal dementia [38]. CXCL12 (SDF-1α) plays a key role in vascular repair mechanisms by mobilizing brain marrow-derived stem cells to the site of lesions [16]. In the treatment of patients with Alzheimer’s disease, medication such as donepezil causes an increase in plasma CXCL12 (SDF-1α) [36]. In the HT group, plasma CXCL-12 (SDF-1α) levels were maintained and this might preserve the cognitive functions of the HT participants by maintaining hematopoietic brain support. 

The significant decline in plasma CXCL5 (RANTES) levels in the control group suggests a deterioration in neuroprotection. It has been proposed that CXCL5 (RANTES) promotes leukocyte infiltration in sites of inflammation and activates T cells [39]. Additionally, CXCL5 (RANTES) also plays an important role in attracting macrophages and leads to the phagocytosis of amyloid-beta plagues [40]. Lower peripheral levels of CXCL5 (RANTES) was observed in people with psychiatric disorders such as schizophrenia [41] and recurrent depressive disorder with suicidal ideation [42]. Recently, CXCL5 (RANTES) has been shown to protect mixed cultures of human neurons and astrocytes from apoptosis [43]. The expression of CXCL5 (RANTES) could have important implications for neuronal function [44]. Hence, low plasma concentrations of CXCL5 (RANTES) could be an important factor in the pathogenesis of psychiatric and neurodegenerative disorders. In this study, the HT group did not show a significant reduction in RANTES. This suggests that HT could play a protective role on inflammation and shows promise in supporting neuroprotection and preventing dementia [44]. 

The significant decline in BDNF in the control group suggests a deterioration in neuronal protection and survival, axonal and dendritic growth. Low peripheral levels of BDNF is associated with lower cognitive test scores and mild cognitive impairment [45] and frailty [46]. In this study, the HT group did not show significant changes in plasma levels of BDNF. Similarly, a 12-week strength training exercise which involves muscle pressing and stretching did not lead to significant changes in BDNF levels in community-dwelling older adults [47]. This could be attributed to the lack of observable change in BDNF due to the short-lived response of BDNF [47]. For example, acute exercise increased the levels of BDNF [48], followed by a significant decrease after a 30-min rest. A 12-week senior brain health exercise program, which includes movements that enhance flexibility, dynamic balance and agility was found to increase BDNF levels in Korean older adults [49]. Therefore, exercises of moderate intensity seem to be more effective in increasing the peripheral levels of BDNF in older adults [46]. In this study, the exercises in the HT program might not be of sufficient intensity to produce an increase in plasma BDNF. 

There were no significant changes in plasma Sgp130 levels in either group. Significant changes in IL-6 levels without changes in Sgp130 levels may indicate that other mechanisms are in place. Additionally, the lack of changes in plasma cortisol levels from baseline to 6-months was not unexpected. In a study of an adult population, elevated cortisol and DHEA levels were observed in response to psychosocial stressors [50]. It is noteworthy that neither of our study groups was exposed to stress. Nicklas et al. compared the effect of moderate-intensity physical activity and health education in older adults and yielded findings similar to our study [51]. As in our study, there was no significant difference in the levels of CRP. Obesity, inflammation and stress are interrelated [52]. Nicklas et al. concluded that an intervention would need to cause weight reduction in order to reduce CRP. In this study, HT was not designed to cause weight reduction and this might explain the lack of significant changes in the level of hs-CRP in the HT group.

This study has addressed a number of the shortcomings in the previous studies on HT. Firstly, this is a RCT comparing the HT treatment group with the waitlist control group, which overcomes the limitations of a single group design [4]. Secondly, we used biological markers to complement the findings obtained from subjective psychological assessments. A range of biomarkers were examined ranging from interleukins, chemokines to hormones. Thirdly, the HT program was designed with contingency plans to address weather issues which might have affected the results in other HT studies [4]. 

Nonetheless, this RCT has several limitations and we thus propose future actions to address these. Firstly, the main limitation of this RCT is the moderate sample size, which indicates the preliminary nature of the results. Although this RCT provides promising data, there were only within-group differences, with no significant between-group differences. These results could be improved with replications using a larger sample size, a longer study duration and higher frequency. Larger sample size may reduce the standard deviations, thus enabling the detection of significant differences between study groups. Another consideration for future research is the “dosage” of the intervention; a diary could be distributed to each participant to note down the exact hours of engaging in HT in-between sessions. Examining the impact of the “dosage” of the intervention could be valuable in pinpointing the optimum hours of exposure to HT to elicit significant effects. Additionally, due to ethical considerations and the exploratory nature of this study, waitlist control design was employed instead of parallel group design. Thus, we were unable to discern the active component of HT. Positive relationships with others was found to be significantly higher in the HT group after intervention, both in our RCT and in previous studies. Future HT trials could control for social interaction using an active control design to discern the exact biopsychosocial mechanisms of HT. Mechanisms of HT intervention on these novel biomarkers were not examined in this study. Future research on the link between HT and improved biomarkers could be performed, for example, a previous study measured phytoncides released from trees and suggested that they could improve biomarkers [53]. 

## 5. Conclusions

The results of this RCT suggest that HT could potentially be useful for reducing inflammation and protecting neuronal functions in healthy elderly adults by reducing plasma IL-6 levels and maintaining plasma CXCL12 (SDF-1α), CXCL5 (RANTES) and BDNF levels. We have provided preliminary biological evidence for the benefits of HT, as well as some hypothesized mechanisms underlying HT that may improve physical and mental health. The significant reductions in plasma IL-6 levels after 6-months in the HT group points to the possibility that elderly adults with inflammatory diseases associated with high IL-6 levels such as depression, dementia, rheumatoid arthritis and cancer could garner benefits from HT. Furthermore, our data contributes to the knowledge gap on how HT might exert hematopoietic brain support and prevent neurodegeneration by maintaining plasma CXCL12 (SDF-1α), CXCL5 (RANTES) and BDNF levels. Further biological studies on the effects of HT are necessary to replicate these findings in larger elderly populations, particularly those with inflammatory disorders and/or high risk of dementia.

## Figures and Tables

**Table 1:** Protocols for horticultural therapy (HT) intervention sessions.

Date (dd/mm/yyyy)	Session	Topic	Activity	Venue
Weekly Sessions				
14/05/2015	1	Introduction	1. TaRA familiarisation 2. Group formation 7/group 3. Indoor Gardening basics	TaRA@JP
21/05/2015	2	Introduction	1. Garden familiarisation 2. Vegetables growing brief 3. Sowing vegetables seedlings	Chinese Garden
28/05/2015	3	Wetland Walk	1. Park amenities familiarization 2. Interpretive walk 3. Reflection	Sungei Buloh Reserve
04/06/2015	4	Introduction	1. Briefing for vegetables maintenance 2. Weeding and fertilizing vegetables plot	Chinese Garden
11/06/2015	5	Nurturing	1. Briefing on pressed flowers 2. Material preparation 3. Make pressed flowers card	TaRA@JP
18/06/2015	6	Nurturing	1. Garden maintenance briefing 2. Weeding, pruning, mulching Arden/vegetables	Chinese Garden
25/06/2015	7	Colour Walk	1. Park amenities familiarization 2. Interpretive walk 3. Reflection	Singapore Botanical Gardens
02/07/2015	8	Nurturing	1. Vegetables maintenance 2. Compost making brief 3. Make compost	Chinese Garden
09/07/2015	9	Harvest and Cook	1. Harvest vegetables 2. Hands on preparation for food 3. Sharing of cooked vegetables	TaRA@JP
16/07/2015	10	Harvest and Cook	1. Seed sowing 2. Herbal plant brief 3. Herbal plants propagation	Chinese Garden
23/07/2015	11	Festive Walk	1. Park amenities familiarization 2. Interpretive walk 3. Reflection	Gardens by the Bay (Cloud Forest/flower dome)
30/07/2015	12	Harvest and Cook	1. Community Garden tour 2. Plant care tips for herbs and plants that will be brought home 3. Reflection	Chinese Garden
Monthly Sessions				
20/08/2015	1	Healing Walk	1. Park amenities familiarization 2. Interpretive walk 3. Reflection	Botanical Garden—Healing Garden
17/09/2015	2	Gardening	1. Briefing 2. Create a culinary garden 3. Maintenance tips	Chinese garden—Culinary garden creation
15/10/2015	3	Nature Walk	1. Park amenities familiarization 2. Interpretive walk 3. Reflection	Gardens by the Bay (Cloud Forest/flower dome)

**Table 2:** Comparison of baseline characteristics between participants in the horticultural therapy and waitlist control arms.

Variables	Active Treatment (*N* = 29)		Waitlist Control (*N* = 30)		Test (*p*-Value)
	Mean (SD)	*N* (%)	Mean (SD)	*N* (%)	
Age	67.21 (4.52)		67.00 (4.18)		*t* = 0.18 (0.86)
Gender					
Male		6 (20.7%)		7 (23.30)	χ^2^ = 0.60 (0.81)
Female		23 (79.3%)		23 (76.7%)	
Years of Formal Education	7.34 (3.89)		7.23 (3.47)		*t* = 0.12 (0.91)
BP (systolic), mmHg	131.93 (18.52)		130.90 (14.56)		*t* = 0.24 (0.81)
BP (diastolic), mmHg	73.93 (11.35)		73.27 (9.14)		*t* = 0.25 (0.81)
Pulse rate, BPM	70.10 (10.84)		67.97 (10.24)		*t* = 0.77 (0.44)
BMI, kg/m^2^	24.37 (3.99)		23.34 (3.31)		*t* = 1.08 (0.28)
Ethnicity					
Chinese		27 (93.1%)		30 (100%)	*F* = 2.14 (0.24)
Indian		2 (6.9%)		0 (0%)	
Others		0 (0%)		0 (0%)	
Employment Status					
Retired		9 (31%)		15 (50%)	*F* = 4.29 (0.33)
Self-employed		1 (3.4%)		0 (0%)	
Full-time worker		0 (0%)		1 (3.3%)	
Part-time worker		8 (27.6%)		7 (23.3%)	
Housewife		11 (37.9%)		7 (23.3%)	
Marital Status					
Never married		0 (0%)		2 (6.7%)	*F* = 3.05 (0.39)
Currently Married		23 (79.3%)		20 (66.7%)	
Divorced		3 (10.3%)		2 (6.7%)	
Widowed		3 (10.3%)		6 (20%)	
Park Visitor					
Yes		16 (55.2%)		15 (50%)	χ^2^ = 0.16 (0.69)
No		13 (44.8%)		15 (50%)	
Perform regular gardening works					
Yes		16 (55.2%)		13 (43.3%)	χ^2^ = 0.83 (0.36)
No		13 (44.8%)		17 (56.7%)	
MoCA	26.34 (2.19)		26.60 (2.31)		*t* = −0.44 (0.67)
SDS	44.69 (3.75)		45.31 (5.33)		*t* = −0.51 (0.61)
SAS	35.14 (2.24)		34.23 (2.53)		*t* = 1.45 (0.15)
Ryff’s Scales of Psychological Well-Being	28.14 (4.87)		27.87 (6.56)		*t* = 1.85 (0.07)
Friendship Scale	11.10 (3.84)		12.07 (4.75)		*t* = −0.85 (0.40)
Satisfaction with Life Scale	79.83 (6.30)		76.47 (7.60)		*t* = 0.18 (0.86)

Abbreviations: MoCA: Montreal Cognitive Assessment, SDS: Zung Self-Rating Depression Scale; SAS: Zung Self-Rating Anxiety Scale; BP: blood pressure; BMI: body mass index.

**Table 3:** Results from the analyses of biomarkers measurements at baseline, 3-months and 6-months post-intervention.

Biomarkers	*p*-Value for Repeated-Measured ANOVA (General Linear Model)	
	Time	Time × Group Interaction
IL-6	0.095	0.41
IL-1β	0.15	0.62
CXCL12 (SDF-1α)	0.007 **	0.20
CXCL5 (RANTES)	0.005 **	0.78
BDNF	0.02 *	0.35
Cortisol	0.35	0.25
DHEA	0.46	0.22
hs-CRP	0.70	0.66
Sgp-130	0.01 *	0.88

BDNF: Brain-derived neurotrophic factor. DHEA: Dehydroepiandrosterone. * denotes *p*-value < 0.05. ** denotes *p*-value < 0.01.

**Table 4:** Results from the analyses of psychometric measures at baseline, 3-months and 6-months post-intervention.

Psychometric Measures	*p*-Value for Repeated-Measured ANOVA (General Linear Model)	
	Time	Time × Group Interaction
MoCA	0.004 **	0.13
SDS	0.53	0.68
SAS	0.03 *	0.81
Ryff’s Scales of Psychological Well-Being	0.86	0.34
Positive Relations with Others Sub-scale	0.14	<0.001 ***
Friendship Scale	0.20	0.10
Satisfaction with Life Scale	0.13	0.64

MoCA: Montreal Cognitive Assessment; SDS: Zung Self-Rating Depression Scale; SAS: Zung Self-Rating Anxiety Scale; Positive Relations with Others Sub-scale is one of the six sub-scales of the Ryff’s Scales of Psychological Well-Being. * denotes *p*-value < 0.05, ** denotes *p*-value < 0.01 and *** denotes *p*-value < 0.001.
